# Relationship Between Weight, Muscle Mass, Cardiorespiratory Endurance, and Likelihood of Dynapenia in Older Adults

**DOI:** 10.1155/jare/5537797

**Published:** 2026-02-18

**Authors:** Mei Chu Chen, Der-Sheng Han, Ke-Vin Chang, Shang-Jyh Chiou

**Affiliations:** ^1^ Department of Physical Medicine and Rehabilitation, Bei-Hu Branch, National Taiwan University Hospital, No 87 Neijiang St., Taipei City, Taiwan, Republic of China, ntuh.gov.tw; ^2^ Department of Physical Medicine and Rehabilitation, College of Medicine, National Taiwan University, No 1, sec. 1, Jen Ai Rd., Taipei City, Taiwan, Republic of China, ntu.edu.tw; ^3^ Department of Health Care Management, National Taipei University of Nursing and Health Sciences, No 365 Ming-te Rd., Taipei City, Taiwan, Republic of China, ntunhs.edu.tw

**Keywords:** cardiorespiratory endurance, dynapenia, elderly health examination, muscle mass, physical fitness examination

## Abstract

**Background:**

In an aging society, the early detection and diagnosis of dynapenia, defined as the *age-associated loss of muscle strength,* are pivotal for preventing or decreasing the deterioration of body functions. This study aims to explore the relationship between dynapenia and nutrition, body fitness, and physical activity.

**Methods:**

This cross‐sectional study surveyed participants at a regional hospital in 2017. The survey utilized the Mini Nutritional Assessment and the International Physical Activity Questionnaire. The body weight, height, appendicular skeletal *muscle*‐mass index (ASMI), calf circumference, balance, lower limb flexibility, muscular endurance, and cardiorespiratory endurance of the participants were measured by trained staff. Additionally, logistic regression models were used to explore the risk factors for dynapenia.

**Results:**

Among the 393 cases, overweight status, obesity, and below‐normal calf circumference were associated with an increased likelihood of dynapenia, with odds ratios (ORs) of 2.15 (*p* = 0.019), 4.15 (*p* = 0.002), and 2.15 (*p* = 0.02), respectively. Conversely, higher ASMI and cardiorespiratory endurance were associated with a decreased likelihood of dynapenia, with ORs of 0.41 (*p* = 0.002) and 0.97 (*p* = 0.001), respectively. Additionally, old age increased the likelihood of dynapenia with an OR of 1.18 (*p* < 0.001). Other variables were not significant.

**Conclusions:**

Controlling body weight and encouraging older adults to engage in physical activities that enhance handgrip strength can reduce the risk of dynapenia. Therefore, promoting self‐care behaviors to reduce the adverse outcomes of dynapenia is crucial for enhancing healthy life in older populations.

## 1. Introduction

Muscle mass and function decline with age [1], and the skeletal muscle involved in mobility, strength, and balance is directly linked to body composition and clinical outcomes (such as frailty) [2, 3]. Muscle function appraisal typically includes measures of strength, muscular performance, or physical performance and is commonly employed in the context of aging.

In 2008, Clark and Manini proposed the term *dynapenia* [4] and found that handgrip and knee‐extensor muscle strengths were strongly related to survival [5]. In 2012, they presented an algorithm for dynapenia screening for individuals above 60 years of age [6, 7]. Henceforth, the most used measurements for dynapenia are handgrip strength and lower extremity muscle performance, such as knee‐extensor muscle strength [8]. Dynapenia refers to the age‐related loss of muscle strength and power and is attributed to factors involving neurological and muscle mechanisms, resulting in muscle weakness [9]. Additionally, low muscle strength is well known to increase the risk of mobility limitations and mortality in older adults [10]. Notably, sarcopenia refers to the loss of both muscle mass and function, while dynapenia specifically describes reduced muscle strength that may not necessarily be accompanied by decreased skeletal muscle mass [11].

The prevalence of dynapenia varies worldwide: 10.3% in older Japanese adults [12], 22.5% in older European adults [13], 25.1% in the older Korean population [14], 69.6% in the older Chinese population (62.3% in men and 72.7% in women) [15], and 24.0% and 21.5% in older Canadian women and men [16], respectively. These variations in prevalence depend on the setting, heterogeneity in methodological aspects, and the few population‐based studies, which limit the ability to make direct comparisons. Different parameters, cut‐off values, and definitions of sarcopenia and dynapenia also lead to varying clinical outcomes. Clinically, patients with dynapenia have a reduced ability to perform daily living activities [17, 18], increased morbidity [19] and mortality [20, 21], and a poorer quality of life [18, 22]. Additionally, patients with dynapenia who also have other comorbidities (such as diabetes, Parkinson’s disease, and anemia) may have increased likelihood of adverse outcomes [23–25].

As people age, muscle mass and strength decline [26]. Longer lifespans and improved healthcare are driving a rise in age‐related muscle loss, threatening mobility and independence while increasing strain on healthcare systems. Addressing this issue is vital for healthy aging and sustainable care. Handgrip strength and gait speed (GS) are positively related to physical activity and overall body function in older individuals. These measures are easy, noninvasive, and significant predictors of mortality in the older population [27]. However, despite the well‐established role of these measures in assessing sarcopenia, dynapenia remains an under‐recognized condition.

Studies focusing on dynapenia are scarce, highlighting the need for a deeper understanding of the mechanisms behind age‐associated strength losses. This study aimed to investigate the relationship between nutrition and physical activity in elderly individuals aged 65 and above with the risk of dynapenia, using a database from a local hospital’s health checkup center. Recognizing and diagnosing dynapenia early can play a crucial role in delaying or preventing the deterioration of body functions.

## 2. Materials and Methods

### 2.1. Setting and Population

This cross‐sectional study involved individuals who participated in the elderly health examination supported by the Taipei City Government and received a check‐up at a regional hospital in northern Taiwan in 2017. The inclusion criteria for the study were age over 65 years and full participation in the functional examination and completion of the questionnaire items. Before the physical examination, trained staff interviewed the participants using the Mini Nutritional Assessment (MNA) and the International Physical Activity Questionnaire (IPAQ). Exclusion criteria included individuals who used pacemakers; had atrial fibrillation/flutter, ventricular bigeminy, infectious diseases, or tumors; were hospitalized; were residents of nursing homes or long‐term care institutions; could not stand stably or complete all functional examinations; or were unable to complete the questionnaire themselves [28]. This study adhered to the ethical principles of the World Medical Association’s Declaration of Helsinki and was approved by the National Taiwan University Hospital Research Ethics Committee (approval No. 201601091RIND).

### 2.2. Variables

#### 2.2.1. Dependent Variable: Dynapenia

Participants were measured the handgrip strength of their dominant hand three times using a Jamar grip dynamometer (Baseline). The best performance was recorded with the subject’s shoulder horizontally adducted, elbow joint flexed at 90°, and forearm in the neutral position. For GS, participants were asked to walk a distance of 5 m at their habitual speed. The walking time was recorded by trained personnel using a handheld stopwatch. The GS was calculated as the 5‐m distance divided by the walking time in seconds (GS = 5 m/walking time in seconds). Dynapenia, or the age‐associated loss of muscle strength, was defined in this study according to the Asian Working Group for Sarcopenia (AWGS) criteria with other research [29], where a person with either low handgrip strength or low GS was classified with dynapenia. Low handgrip strength was defined as less than 28 kg for males and less than 18 kg for females. Low GS was defined as less than 1.0 m/s for both genders [30].

#### 2.2.2. Independent Variable: Functional and Physical Status

Functional and physical status measurements included height, weight, appendicular skeletal muscle mass index (ASMI), balance, flexibility, muscular endurance, and cardiorespiratory endurance. ASMI = appendicular skeletal muscle mass (kg)/height^2^ (meter)^2^ was measured using the multiple‐frequency bioelectrical impedance analysis (MFBIA) (InBody 720, Biospace, Seoul, Korea, with four poles and eight contact electrodes and electrical phase frequency at 1, 5, 50, 250, 500, and 1000 kHz.), with values of less than 7.0 kg/m^2^ for males and less than 5.7 kg/m^2^ for females considered low muscle mass followed AWGS suggestions [20]. Balance was assessed using the forward functional reach test. Participants stood with their dominant arm extended parallel to a wall‐mounted yardstick positioned at acromion height. They were instructed to avoid touching the wall, extend the elbow with the shoulder flexed to 90°, form a fist, and maintain the posture for 3 s. The location of the third metacarpal was recorded at rest (Position 1) and again during maximal forward reach without stepping or contacting the wall (Position 2). Each participant completed two trials, and functional reach was calculated as the mean difference between Positions 2 and 1 from the final valid trials. A reach distance of 25.4 cm or greater for that is held at least 2 s corresponded to a low risk of falls [31]; 15.24–25.4 cm indicated a moderate risk; and less than 15.24 cm indicated a significant risk. Flexibility was measured using the chair sit‐and‐reach test, performed twice, with the best value recorded. Fingers just touch the toes (0 score). If fingers do not reach the toes, the distance is measured (negative score); if fingers go past the toes, the distance is measured (positive score). The normal cut‐off points were ≥ −10 cm for males and ≥ −5 cm for females [32]. Muscular endurance was assessed using the 30‐s chair stand test, which measures the number of times a participant can rise to a standing position from a seated position within 30 s, with the cut‐off being ≥ 8 times for healthy adults [33–35]. Cardiorespiratory endurance was measured using the 2‐min step test, where participants tread in place for 2 min, lifting their right knee to a taped midpoint between the hip and kneecap, with each successful lift counted. The cut‐off points for normal adults are ≥ 65 steps [36]. These parameters were measured by trained physical therapists.

#### 2.2.3. Independent Variable: MNA and IPAQ Questionnaire

##### 2.2.3.1. MNA

The MNA contains 18 items divided into two parts. The nutrition screening combined with the assessment yields a maximum score of 30, with categories for normal nutritional status (30–24), risk of malnutrition (17–23.5), and malnourished (0–16) [37].

##### 2.2.3.2. IPAQ

We used the Chinese version of the IPAQ to evaluate physical activities. The IPAQ contains seven items assessing activities from vigorous to light intensity over the past 7 days. Physical activity was quantified in metabolic equivalent (MET) task minutes per week (MET‐min/wk), with categories for high (> 3000 METs), medium (600–2999 METs), and low (< 600 METs) levels of physical activity [38].

##### 2.2.3.3. Covariables

Covariables included sex, age (65–74, and > 75), height, weight, body mass index (BMI) (catalogized as Normal (18.5–23.9), Overweight (24.0–26.9), and Obesity (≧ 27.0)) [39], and calf circumference. The weight and height were measured through the automatic machine SUPER‐VIEW (HW‐586A), and BMI was calculated as weight (kg)/height^2^ (meter)^2^. Please note that age is a continuous variable, and we have categorized it into 65–74 (young old), 75–84 (middle‐old), and 85 years and over (oldest old) [40], combining the middle‐old and oldest‐old groups due to small sample sizes in the oldest‐old group in this study. Calf circumference (cm) was measured with a flexible measuring tape at the point of greatest circumference of the leg in the standing position. The cut‐points for normal calf circumference are greater than or equal to 34 cm (males) or 33 cm (females) [41].

##### 2.2.3.4. Statistical Analysis

Descriptive analysis (number with % and mean with standard deviation) was used to display the distribution of demographics, physical and functional status, and MNA and IPAQ scores for the study population. Differences in distributions were examined by comparing sex or (with or without) dynapenia using the chi‐square or the Mann–Whitney *U* test due to outliers affecting the normal distributions (as determined by the Kolmogorov–Smirnov test). A simple logistic regression model was used to explore the relationship between the risk of dynapenia and the determining factors first, followed by multiple logistic regression using different model constructions. As handgrip strength and GS were used to define dynapenia, these variables were excluded from the logistic regression model. Model construction began with age (continuous), sex, MNA (normal, or at‐risk group), and IPAQ (high, medium, or low group) in model 1. Subsequent models gradually included additional variables: BMI (catalog as normal, overweight, and obesity group) in model 2; ASMI (continuous) in model 3; and calf circumference (normal, and below the normal group), balance (normal, and at risk group), flexibility (in the normal and below the normal group), muscular endurance, and cardiorespiratory endurance in model 4. Calf circumference, BMI, ASMI, balance, flexibility, muscular endurance, cardiorespiratory endurance, MNA, and IPAQ were initially assessed as continuous variables. For interpretability, these measures are often categorized into groups (e.g., normal vs. abnormal or at risk vs. not at risk) to facilitate the calculation of odds ratios (ORs) for dynapenia. ORs are widely used because they provide a straightforward way for readers to understand relative risk. However, the small number of participants classified as “at risk” raised concerns about inflated effect estimates from categorical comparisons. To mitigate this, in model 4, we retained muscular endurance, ASMI, and cardiorespiratory endurance as continuous variables. This approach reduced the risk of overestimation and yielded a more stable evaluation of their association with dynapenia. For sensitivity analysis, we ran model 5 using MNA, calf circumference, balance, and flexibility as continuous variables. Additionally, the models did not include any interaction terms based on the independence hypothesis, as including interaction terms may render the results difficult to explain. All statistical analyses were conducted using SAS software version 9.3.1 (SAS Institute, Cary, NC, USA) and Statistical Package for the Social Sciences software (version 20.0; SPSS Inc., Chicago, IL, USA). The significance level was set at a *p* value of less than 0.05, and the goodness‐of‐fit (Nagelkerke pseudo‐R2, and AIC) in the full model are reported.

## 3. Results

This study included 393 cases, with an average age of 72.5 years and predominantly female participants. Regarding functional status, the average handgrip strength, GS, and ASMI were 20 kg, 1.17 m/s, and 7.0 kg/m^2^, respectively. The elderly had 72% and 38.4% values for calf circumference and BMI in the normal range, respectively. Acceptable levels in balance, flexibility, muscular endurance, and cardiorespiratory endurance were observed in 92.4%, 88.3%, 98.2%, and 95.2% of participants, respectively. Normal scores were achieved by 93.1% in the MNA and 54.2% in the median level of the IPAQ (Table 1).

**TABLE 1:** The demography and items distributions of participants (*N* = 393).

Variables	N (%)/Mean ± SD
*Demography Sex*	
Male	173 (44)
Female	220 (56)
Age groups	72.53 ± 6.07
65–74	248 (63.1)
> 75	145 (36.9)
Height (cm)	159.04 ± 7.98
Weight (kg)	63.58 ± 9.67

*Physical exam/functional status*	
Handgrip strength (kg) Low muscle strength	20.00 ± 8.46 259 (65.9%)
Gait speed (m/s)	1.17 ± 0.34
Low gait speed	129 (32.8%)
Calf circumference (cm)^^^	34.89 ± 3.07
Normal range	282 (71.8)
BMI (kg/m^2^)^#^	25.10 ± 3.09
Normal (18.5–23.9)	151 (38.4)
Overweight (24.0–26.9)	143 (36.4)
Obesity (≧ 27.0)	99 (25.2)
ASMI (kg/m^2^)	6.99 ± 1.06
Normal	355 (90.3)
Balance (cm)^@^	35.84 ± 7.27
No risk of fall	363 (92.4)
Flexibility (cm)^@@^	4.72 ± 11.67
Normal	347 (88.3)
Muscular endurance (times)^@@@^	16.80 ± 5.78
Normal	386 (98.2)
Cardiorespiratory endurance (steps)^@@@@^	105.00 ± 21.16
Normal	374 (95.2)
MNA (max 30)^&^	27.16 ± 2.16
Normal	366 (93.1)
At risk	27 (6.9)
IPAQ (METs‐min/week)^&&^	2701.44 ± 1845.51
High	140 (35.6)
medium	213 (54.2)
Low	40 (10.2)

^^^Calf circumference, the normal: male ≧ 34 cm, female ≧ 33 cm.

^#^BMI: Body mass index.

^##^ASMI (kg/m^2^): Appendicular skeletal muscle mass index, male ≦ 7.0 kg/m^2^.

female ≦ 5.7 kg/m^2^.

^@^Balance, no risk of fall > 25.5 cm, risk of fall < 25.4 cm.

^@@^Flexibility (cm), in the normal: male ≧ 10 cm, female ≧ −5 cm.

^@@@^Muscular endurance, normal ≧ 8 times.

^@@@@^Cardiorespiratory endurance (stepping up and down on a step for two minutes): normal ≧ 65 steps.

^&^MNA: Mini‐Nutritional Assessment (0–30), normal > 24, risk < 23.5.

^&&^IPAQ: International Physical Activity Questionnaire, high > 3000 METs‐min/week, median 600–2999 METs‐min/week, low:< 600 METs‐min/week.

The study group included 173 males and 220 females. Table 2 presents the demographic and functional status distribution by sex. Male participants were slightly older and demonstrated superior handgrip strength (*p* < 0.001), GS (*p* = 0.022), ASMI (*p* < 0.001), balance (*p* < 0.001), muscular endurance (*p* < 0.001), and MNA scores (*p* = 0.012). In contrast, females had greater calf circumference (*p* = 0.04), BMI (*p* = 0.028), flexibility (*P* < 0.001), and IPAQ results. Significant differences between males and females were observed in height, weight, handgrip strength, balance, and MNA scores.

**TABLE 2:** The demography and items distributions of participants in different sex.

Variables	Male (*n* = 173)	Female (*n* = 220)	*p*
	N (%)/Mean ± SD	N (%)/Mean ± SD	
Age groups	73.03 ± 6.38	72.13 ± 5.79	0.144
65–74	105 (60.7)	143 (65)	0.380
> 75	68 (39.3)	77 (35)	
Height (cm)	165.41 ± 5.52	154.04 ± 5.73	< 0.001
Weight (kg)	69.68 ± 8.50	58.79 ± 7.63	< 0.001
*Physical exam/functional status*			
Handgrip strength(kg)	20.94 ± 6.77	14.55 ± 4.92	< 0.001
Gait speed (m/s)	1.22 ± 0.36	1.14 ± 0.33	0.006
Calf circumference (cm)^^^	35.24 ± 2.80	34.61 ± 3.25	0.021
Normal	109 (63.0)	142 (64.5)	0.752
BMI (kg/m^2^)^#^	25.49 ± 3.02	24.80 ± 3.11	0.024
Normal	56 (32.4)	96 (43.6)	0.029
Overweight	68 (39.3)	74 (33.6)	
Obesity	49 (28.3)	50 (22.8)	
ASMI (kg/m^2^)	7.84 ± 0.61	6.32 ± 0.82	< 0.001
normal	168 (95.4)	190 (86.4)	0.003
Balance (cm)^@^	38.82 ± 7.29	33.49 ± 6.34	< 0.001
No risk of fall	167 (96.5)	196 (89.1)	0.006
Flexibility (cm)^@@^	2.08 ± 12.02	6.79 ± 10.99	< 0.001
Normal	148 (85.5)	193 (87.7)	0.527
Muscular endurance (times)^@@@^	18.09 ± 5.94	15.78 ± 5.45	< 0.001
Normal	170 (98.3)	209 (95.0)	0.083
Cardiorespiratory endurance (steps)^@@@@^	105.40 ± 21.18	104.69 ± 21.18	0.629
Normal	166 (96.0)	208 (94.5)	0.518
MNA (max 30)^&^	27.47 ± 2.03	26.92 ± 2.24	0.013
Normal	166 (96.0)	200 (90.9)	0.05
At risk	7 (4.0)	20 (9.1)	
IPAQ (METs‐min/week)^&&^	2615.27 ± 1911.4	2969.20 ± 1793.5	0.211
High	59 (34.1)	81 (36.8)	0.048
medium	90 (52.0)	122 (55.5)	
Low	24 (13.9)	17 (7.7)	

*Note: p* from chi‐square test, or nonparametric *p* value from Mann–Whitney *U* test.

^^^Calf circumference, the normal: male ≧ 34 cm, female ≧ 33 cm.

^#^BMI: Body mass index.

^##^ASMI (kg/m^2^): Appendicular skeletal muscle mass index, male ≦ 7.0 kg/m^2^.

female ≦ 5.7 kg/m^2^.

^@^Balance, no risk of fall > 25.5 cm, risk of fall < 25.4 cm.

^@@^Flexibility (cm), in the normal: male ≧ −10 cm, female ≧ −5 cm.

^@@@^Muscular endurance, normal ≧ 8 times.

^@@@@^Cardiorespiratory endurance (stepping up and down on a step for two minutes): normal  65 steps.

^&^MNA: Mini‐Nutritional Assessment (0–30), normal > 24, risk < 23.5.

^&&^IPAQ: International Physical Activity Questionnaire, high > 3000 METs‐min/week, median 600–2999 METs‐min/week, low: < 600 METs‐min/week.

Table 3 presents the comparison of the characteristics of participants without dynapenia to those with dynapenia. The dynapenia group predominantly comprised older females with significantly lower handgrip strength, GS, ASMI, balance, and cardiorespiratory endurance compared to the no‐dynapenia group.

**TABLE 3:** The demography and items distributions of participants in different groups.

	No dynapenia (*n* = 108)	Dynapenia (*n* = 285)	*p* value
	N (%)/Mean ± SD	N (%)/Mean ± SD	
*Demography*			
Sex			< 0.001^∗∗∗^
Male	69 (64%)	104 (36%)	
Female	39 (36%)	181 (64%)	
Age	69.31 ± 4.03	73.75 ± 6.27	< 0.001^∗∗∗^
Height (cm)	162.97 ± 7.06	157.55 ± 7.81	< 0.001^∗∗∗^
Weight (kg)	65.49 ± 8.94	62.86 ± 9.85	0.004^∗^

*Physical exam/functional status*			
Handgrip strength (kg)	28.62 ± 7.35	16.74 ± 6.29	< 0.001^∗∗∗^
Gait speed (m/s)	1.37 ± 0.24	1.10 ± 0.35	< 0.001^∗∗∗^
Calf circumference (cm)^^^	35.45 ± 2.63	34.67 ± 3.20	0.020^∗^
Normal range	84 (77.7%)	198 (69.5%)	0.103
BMI (kg/m^2^)^#^	24.63 ± 2.83	25.28 ± 3.17	0.062
Normal	49 (45.4%)	102 (35.8%)	0.207
Overweight	36 (33.3%)	107 (37.5%)	
Obesity	23 (21.3%)	76 (26.7%)	
ASMI (kg/m^2^)	7.47 ± 1.18	6.81 ± 0.95	< 0.001^∗∗∗^
normal	105 (97.2)	250 (87.7)	0.004
Balance (cm)^@^	39.75 ± 5.80	34.35 ± 7.22	< 0.001^∗∗∗^
no risk of fall	107 (99.1%)	256 (89.8%)	0.002^∗∗^
Flexibility (cm)^@@^	7.50 ± 12.09	3.66 ± 11.36	0.004^∗∗^
Normal	99 (91.7%)	248 (87.0%)	0.201
Muscular endurance (times)^@@@^	19.59 ± 6.16	15.74 ± 5.26	< 0.001^∗∗∗^
Normal	108 (100%)	278 (97.5%)	0.100
Cardiorespiratory endurance (stpes)^@@@@^	113.80 ± 19.45	101.67 ± 20.85	< 0.001^∗∗∗^
Normal	108 (100%)	266 (93.3%)	0.006^∗∗^
MNA (max 30)^&^	27.45 ± 1.97	27.05 ± 2.23	0.144
Normal	105 (97.2%)	261 (91.6%)	0.048^∗^
At risk	3 (2.8%)	24 (8.4%)	
IPAQ (METs‐min/week)^&&^	2763.02 ± 1883.64	2678.10 ± 1833.67	0.763
High	36 (33.3%)	104 (36.5%)	0.731
medium	62 (57.4%)	151 (53.0%)	
Low	10 (9.3%)	30 (10.5%)	

*Note:* from chi‐square test, or nonparametric *p* value from Mann–Whitney *U* test.

^∗^ *p* < 0.05,^∗∗^ *p* < 0.01,^∗∗∗^ *p* < 0.001.

^^^Calf circumference, the normal: male ≧ 34 cm, female ≧ 33 cm.

^#^BMI: Body mass index.

^##^ASMI (kg/m^2^): Appendicular skeletal muscle mass index, male ≦ 7.0 kg/m^2^.

female ≦ 5.7 kg/m^2^.

^@^Balance, no risk of fall > 25.5 cm, risk of fall < 25.4 cm.

^@@^Flexibility (cm), in the normal: male ≧ −10 cm, female ≧−5 cm.

^@@@^Muscular endurance, normal ≧ 8 times.

^@@@@^Cardiorespiratory endurance (stepping up and down on a step for two minutes): normal ≧ 65 steps.

^&^MNA: Mini‐Nutritional Assessment (0–30), normal > 24, at risk < 23.5.

^&&^IPAQ: International Physical Activity Questionnaire, high > 3000 METs‐min/week, median 600–2999 METs‐min/week, low: < 600 METs‐min/week.

nonparametric *p* value from Mann–Whitney *U* test.

Table 4 shows the results of simple logistic regression analyses to determine the likelihood of dynapenia based on various factors. Older age or being in the balance group at risk of falls increased the likelihood of dynapenia. Conversely, being male or having higher ASMI, muscular endurance, or cardiorespiratory endurance was associated with a decreased likelihood of dynapenia.

**TABLE 4:** The relationship between dynapenia with determining factors from simple logistic regression.

Variable	OR	95% CI of OR	*p*
Age (65–74, ref)			
> 75	5.20	2.87–9.41	< 0.001^∗∗∗^
*Sex (female, ref)*			
Male	0.33	0.21–0.52	< 0.001^∗∗∗^
Height (cm)	0.91	0.88–0.94	< 0.001^∗∗∗^
Weight (kg)	0.97	0.95–0.99	0.017^∗^
*Calf circumference (normal, ref)*			
Below the normal	1.54	0.92–2.58	0.104

*BMI (kg/* *m* ^2^ *) (normal, ref)*			
Overweight	1.43	0.86–2.37	0.17
Obesity	1.59	0.89–2.83	0.12
ASMI (kg/m^2^)	0.54	0.42–0.68	< 0.001^∗∗∗^

Balance (> 25.5 cm, ref)			
At risk	12.12	1.63–90.12	0.015^∗^

Flexibility (in the normal, ref)			
Below the normal	1.64	0.76–3.53	0.204
Muscular endurance (times)	0.89	0.85–0.93	< 0.001^∗∗∗^
Cardiorespiratory endurance (steps)	0.97	0.96–0.98	< 0.001^∗∗∗^

*MNA (normal, ref)*			
At risk	3.22	0.95–10.92	0.061

*IPAQ (high, ref)*			
Medium	0.84	0.52–1.36	0.486
Low	1.04	0.46–2.33	0.927

^∗^ *p* < 0.05,^∗∗^ *p* < 0.01,^∗∗∗^ *p* < 0.001.

Table 5 presents the results of multivariable logistic regression analyses investigating the relationship between dynapenia and various factors. Model 1 included demographic factors, MNA, and IPAQ; model 2 added BMI; model 3 included ASMI; and model 4 incorporated calf circumference, balance, flexibility, muscular endurance, and cardiorespiratory endurance. The final model revealed that older age (OR: 1.2, *p* < 0.001), being overweight or obese (OR: 2.15 or 4.15), and having below‐normal calf circumference (OR: 2.15, *p* = 0.02) significantly increased the likelihood of dynapenia. Higher ASMI (OR: 0.41, *p* = 0.002) and greater cardiorespiratory endurance (OR: 0.97, *p* = 0.001) were associated with a reduced likelihood of dynapenia.

**TABLE 5:** The relationship between dynapenia with determining factors adjusted in different models from logistic regression.

variables	Model 1		*p*	Model 2		*p*	Model 3		*p*	Model 4		*p*
	OR	95% CI of OR		OR	95% CI of OR		OR	95% CI of OR		OR	95% CI of OR	
Age	1.19	1.13–1.26	< 0.001^∗∗∗^	1.20	1.38–1.27	< 0.001^∗∗∗^	1.2	1.13–1.26	< 0.001^∗∗∗^	1.18	1.11–1.25	< 0.001^∗∗∗^
Sex (female, ref)	0.24	0.14–0.40	< 0.001^∗∗∗^	0.21	0.12–0.36	< 0.001^∗∗∗^	0.67	0.26–1.69	0.39	0.81	0.30–2.21	0.69

*MNA (normal, ref)*												
At risk	2.90	0.78–10.83	0.11	2.68	0.70–10.18	0.15	2.78	0.71–10.85	0.14	2.46	0.60–10.03	0.21
*IPAQ (high, ref)*												
Medium	0.74	0.43–1.27	0.27	0.69	0.40–1.20	0.19	0.68	0.39–1.20	0.18	0.57	0.31–1.03	0.61
Low	1.47	0.58–3.74	0.42	1.27	0.49–3.27	0.63	1.22	0.50–3.19	0.68	0.8	0.29–2.21	0.67
BMI (kg/m^2^) (normal, ref)												
Overweight (24.0–26.9)				1.77	0.98–3.19	0.06	2.06	1.12–3.79	0.02^∗^	2.15	1.14–4.09	0.019^∗^
Obesity (≧ 27.0)				2.24	1.14–4.40	0.02^∗^	4.31	1.86–9.98	0.001^∗∗^	4.15	1.71–10.05	0.002^∗∗^
ASMI (kg/m^2^)							0.45	0.27–0.76	0.003^∗^	0.41	0.23–0.73	0.002^∗∗^

*Calf circumference (normal, ref)*												
Below the normal										2.15	1.10–4.19	0.02^∗^
Balance (> 25.5 cm, ref)												
At risk(< 25.4 cm)										2.99	0.37–23.99	0.3
Flexibility (in the normal, ref)												
Below the normal										2.04	0.74–5.62	0.17
Muscular endurance (times)										0.98	0.93–1.03	0.41
Cardiorespiratory endurance (steps)										0.97	0.96–0.99	0.001^∗∗^

*Note:* Model 1: adjusted by age, sex, MNA, IPAQ, Model 2: Model 1 + BMI, Model 3: Model 2 + ASMI, Model 4: Model 3 + Calf circumference, balance, flexibility, Muscular endurance, Cardiorespiratory endurance, the goodness‐of‐fit (Nagelkerke pseudo‐R2: 0.416, and AIC = 2 × 11 + 328.807 = 350.87) in the full model is reported, the goodness‐of‐fit (Nagelkerke pseudo‐R2: 0.336, and AIC = 2 × 6 + 358.086 = 370.086) in the model 3 is reported, the goodness‐of‐fit (Nagelkerke pseudo‐R2: 0.303, and AIC = 2 × 5 + 369.804 = 379.804) in the model 2 is reported, the goodness‐of‐fit (Nagelkerke pseudo‐R2: 0.283, and AIC = 2 × 4 + 376.468 = 384.468) in the model 1 is reported.

^∗^ *p* < 0.05,^∗∗^ *p* < 0.01,^∗∗∗^ *p* < 0.001.

MNA, calf circumference, balance, and flexibility are the continuous variables given in Table S2 (supplemental file). The results are similar: MNA and calf circumference are not significant, while higher balance and higher flexibility have slightly lower likelihoods of dynapenia with ORs of 0.949 (95% CI: 0.903–0.998, *p* = 0.04) and 0.971 (95% CI: 0.933–0.997, *P* = 0.032), respectively.

## 4. Discussion

This study revealed that older age, higher BMI, lower ASMI, below‐normal calf circumference, and lower cardiorespiratory endurance are factors significantly associated with an increased likelihood of dynapenia. The majority of the participants were in the normal range for nutritional assessment and engaged in moderate levels of physical activity; therefore, the MNA and IPAQ were not significantly associated with dynapenia. The high prevalence of dynapenia in this study group should alert authorities to pay more attention to this emerging condition in older populations and provide screening and effective interventions to slow down the progression of dynapenia in aging societies.

Dynapenia is a common but often under‐recognized age‐associated condition among the older population. Aging is accompanied by a certain degree of muscle mass loss. Lean muscle mass is an indicator of strength, muscle function, and metabolism, and decreased lean muscle mass increases the risk of dynapenia; its long‐term impacts (such as falls, sarcopenia or even mortality) need to be considered as well [42, 43]. Most older adults may not be aware of their muscle mass decrease initially, especially since estimating the muscle mass index through ASMI requires equipment (such as dual‐energy X‐ray absorptiometry) available in institutions. Authorities should promote regular physical examinations for the elderly, including mandatory muscle mass index assessments, and provide financial subsidies for these examinations as an incentive to draw attention to muscle mass decrease and its prevention.

The emergence of the obesity epidemic in older adults can increase the risk of chronic diseases (such as diabetes) and lead to several adverse outcomes, including physical function decline, morbidity, and institutionalization [44]. In this study, being overweight or obese was associated with an increased likelihood of dynapenia, which aligns with other studies [12, 45, 46]. While no clear explanation exists for why obesity increases the likelihood of dynapenia, a possible explanation is the complication in muscle mass, bone, and obesity in older individuals, leading to different syndromes ranging from osteopenia and sarcopenia to obesity. Additionally, one study revealed that weight‐loss intervention combined with exercise should be advised for obese individuals with sarcopenia or dynapenia to avoid undesirable losses in muscle and bone [44]. Further research needs to classify individuals with dynapenic or sarcopenic obesity because older individuals with both conditions (dynapenia/obesity) would have more impaired physical function than those with dynapenia or obesity alone and may need different interventions.

Calf circumference is a predictor of skeletal muscle mass, and AWGS uses calf circumference in the criteria for sarcopenia diagnosis. Measurements of calf circumference can provide normal muscle mass information, and low values reflect decreased muscle mass with limited physical activities. In this study, higher calf circumference was associated with a decreased likelihood of dynapenia, which concurs with other studies on sarcopenia [47]. Most importantly, we may consider using handgrip strength and calf circumference as screening tools for dynapenia [48] or as feasible alternatives for assessing nutritional status and reminding older individuals to be aware of their muscle strength and mass to prevent the risk of dynapenia.

Cardiorespiratory endurance is needed for sustained physical activity, and low cardiorespiratory fitness and muscle strength are independently associated with an increased risk of cardiovascular diseases. Our study suggested that higher cardiorespiratory endurance slightly decreased the likelihood of dynapenia. Other studies have observed similar results but were based on specific populations with more complex measurements [49]. Encouraging older adults to engage in moderate‐intensity exercise (such as dancing, walking, or swimming) can benefit their heart, lungs, and muscles, and decrease the risk of diseases and dynapenia.

In this study, dynapenia was found to be associated with distinct sex‐specific risk factors (stratified analysis in the supporting file Table S1). Despite age, among males, obesity status, reduced calf circumference, lower ASMI, and lower cardiorespiratory endurance significantly increased the likelihood of dynapenia. In contrast, among females, overweight, and a lower ASMI emerged as the primary predictors. These findings underscore the importance of developing sex‐sensitive intervention strategies to effectively address dynapenia in aging populations.

Muscle weakness can lead to greater health problems, such as frailty syndrome, falls, and fractures, which are common concerns for older people [44]. Dynapenia describes age‐related loss of muscle strength. A study in Brazil identified age, physical activity level, sedentary behavior, and nutritional status as factors associated with dynapenia [50]. Lifestyle interventions, such as nutrition and physical activity, are two important behavioral factors in maintaining muscle strength [15, 44, 51]. Both dynapenia and sarcopenia are strongly associated with older adults’ activities of daily living, frailty, falls, and fear of falling, which can lead to disability and mortality. Therefore, evaluating muscular strength rather than muscle mass is crucial [9, 10]. Additionally, longitudinal or cohort studies will help understand the impact of dynapenia and alter body composition on disease presentation and progression. Targeting future interventions aimed at strength preservation in the elderly population is pivotal to preventing healthy life expectancy loss in an aging society.

### 4.1. Limitation

Measurements in this study come from individuals who participated in the physical examination at the institution, suggesting they may have been more aware of their health status. Therefore, while nutrition status and physical activities are important factors associated with dynapenia in other studies, the slight differences from MNA and IPAQ in our study may be explained by selection bias. These findings, therefore, have limited extrapolation to other settings. Regarding the classification of dynapenia, there was no clear operational definition for screening in the early days. In 2014, AWGS introduced the thresholds for handgrip strength and habitual GS for the Asian population. In 2019, the AWGS in 2019 released a revised consensus report that included the term “possible sarcopenia” to emphasize early detection and intervention [20]. As dynapenia is considered a precursor to sarcopenia, both conditions should be analyzed simultaneously and evaluated using consistent criteria, although some studies only use handgrip strength [14], GS [52], and/or muscle mass [53]. Additionally, the cut‐off points for handgrip strength in defining dynapenia vary between studies, ranging from 26 kg to 33 kg for men and from 16 to 21 kg for women [54]. Different cut‐offs in dynapenia criteria may affect the results. Furthermore, this study did not collect data on health behaviors, diet, family history, or medication information, which could clarify the association with the likelihood of dynapenia. As a cross‐sectional survey, it is also challenging to explore the cause–effect relationship between these factors and dynapenia. Despite these limitations, this study still provides important findings that confirm skeletal muscle mass and lower‐body strength as crucial factors, highlighting the need to focus on dynapenia in the older population.

## 5. Conclusion

In this study, we found dynapenia in older adults is strongly influenced by cardiorespiratory fitness and body composition, addressing risk factors like obesity, overweight status, and low calf circumference while promoting protective factors such as higher muscle mass and better cardiorespiratory endurance. Effective prevention requires actionable strategies such as weight control and endurance‐promoting activities to enhance cardiovascular capacity. With increasing age, physical activity decreases, leading to potential health problems in older adults. Controlling body weight and encouraging older adults to engage in physical activities to enhance handgrip strength can reduce the risk of dynapenia. Therefore, promoting self‐care behaviors to reduce the adverse outcomes of dynapenia is crucial for improving the quality of life among older adults. In addition, dynapenia in older adults is driven by sex‐specific risk factors highlighting the need for tailored, sex‐sensitive intervention strategies.

## Funding

The authors received no specific funding for this work.

## Ethics Statement

This study adhered to the ethical principles of the World Medical Association’s Declaration of Helsinki and was approved by the National Taiwan University Hospital Research Ethics Committee (approval no. 201601091RIND).

## Consent

Complete written informed consent was obtained from the patient for the publication of this study.

## Conflicts of Interest

The authors declare no conflicts of interest.

## Supporting Information

Two tables are in the supplemental file including: Table S1, the relationship between dynapenia with determining factors from multiple logistic regression stratified by sex, and Table S2, the relationship between dynapenia with determining factors from multiple logistic regression comparing some variables using continuous scale.

## Supporting information


**Supporting Information** Additional supporting information can be found online in the Supporting Information section.

## Data Availability

The datasets generated and/or analyzed during the present study are not publicly available due to ethical restrictions. Additionally, the supporting data cannot be made openly available.
