# Supplementary material for: Relationship Between Weight, Muscle Mass, Cardiorespiratory Endurance, and Likelihood of Dynapenia in Older Adults
Source: J Aging Res. 2026 Feb 18;2026:5537797. doi: 10.1155/jare/5537797 (PMC12914332; doi:10.1155/jare/5537797)
Supplement: Supplementary file 1 — Supporting Information Additional supporting information can be found online in the Supporting Information section. [file JARE-2026-5537797-s001.docx]

Table S1 the relationship between dynapenia with determining factors from multiple logistic regression stratified by sex

|  | female |  |  |  | male |  |  |  |
| --- | --- | --- | --- | --- | --- | --- | --- | --- |
| variables | OR | *Up* | *lo* | *P* | OR | *Up* | *lo* | *P* |
| Age | 1.174 | 1.067 | 1.292 | 0.001 | 1.219 | 1.114 | 1.334 | <0.001 |
| MNA (normal, ref vs At risk) | 1.487 | 0.305 | 7.250 | 0.624 | * | 0.000 | . | 0.999 |
| IPAQ (high, ref) |  |  |  | 0.219 |  |  |  | 0.292 |
| Median | 0.481 | 0.204 | 1.133 | 0.094 | 0.510 | 0.207 | 1.255 | 0.143 |
| Low | 1.059 | 0.108 | 10.417 | 0.961 | 0.454 | 0.118 | 1.746 | 0.251 |
| BMI (normal, ref) |  |  |  | 0.063 |  |  |  | 0.104 |
| Overweight (24.0 - 26.9) | 3.025 | 1.132 | 8.089 | 0.027 | 1.826 | 0.690 | 4.836 | 0.225 |
| Obesity (≧27.0) | 2.655 | 0.764 | 9.227 | 0.124 | 4.180 | 1.118 | 15.627 | 0.034 |
| ASMI (kg/m^2^) | 0.426 | 0.185 | 0.980 | 0.045 | 0.430 | 0.186 | 0.995 | 0.049 |
| Calf circumference (normal, ref vs Below the normal) | 0.781 | 0.303 | 2.018 | 0.611 | 4.497 | 1.656 | 12.211 | 0.003 |
| Flexibility (in the normal, ref vs Below the normal) | * | 0.000 | . | 0.998 | 0.761 | 0.054 | 10.673 | 0.839 |
| Balance (＞25.5cm, ref vs At risk(＜25.4cm) | 7.468 | 0.810 | 68.811 | 0.076 | 1.521 | 0.352 | 6.585 | 0.575 |
| Muscular endurance (times) | 1.010 | 0.926 | 1.101 | 0.825 | 0.941 | 0.864 | 1.025 | 0.162 |
| Cardiorespiratory endurance (steps) | 0.982 | 0.958 | 1.006 | 0.140 | 0.963 | 0.937 | 0.989 | 0.005 |

BMI: Body Mass Index

ASMI: Appendicular skeletal muscle mass index

MNA: Mini*-*Nutritional Assessment

IPAQ: International Physical Activity Questionnaire

* Cell numbers are too small, leading to wide confidence intervals and potential overestimation

Table S2 the relationship between dynapenia with determining factors from multiple logistic regression comparing some variables using continuous scale

|  | Model 4 | | |  | Model 5 | | |
| --- | --- | --- | --- | --- | --- | --- | --- |
| variables | OR | 95% CI of OR | *P* |  | OR | 95% CI of OR | *P* |
| Age | 1.18 | 1.11~1.25 | <0.001 | Age | 1.170 | 1.101~1.244 | <0.001 |
| Sex (female, ref) | 0.81 | 0.30~2.21 | 0.69 | Sex (female, ref) | 0.828 | 0.294~2.332 | 0.720 |
| MNA (normal, ref vs At risk) | 2.46 | 0.60~10.03 | 0.21 | MNA* | 0.978 | 0.851~1.125 | 0.760 |
| IPAQ (high, ref) |  |  |  | IPAQ (high, ref) |  |  |  |
| Medium | 0.57 | 0.31~1.03 | 0.61 | Medium | 0.586 | 0.319~1.075 | 0.084 |
| Low | 0.8 | 0.29~2.21 | 0.67 | Low | 0.854 | 0.311~2.343 | 0.759 |
| BMI (normal, ref) |  |  |  | BMI (normal, ref) |  |  |  |
| Overweight (24.0 - 26.9) | 2.15 | 1.14~4.09 | 0.019 | Overweight (24.0 - 26.9) | 1.973 | 1.029~3.784 | 0.041 |
| Obesity (≧27.0) | 4.15 | 1.71~10.05 | 0.002 | Obesity (≧27.0) | 3.221 | 1.289~8.054 | 0.012 |
| ASMI (kg/m^2^) | 0.41 | 0.23~0.73 | 0.002 | ASMI (kg/m^2^) | 0.423 | 0.235~0.760 | 0.004 |
| Calf circumference (normal, ref vs Below the normal) | 2.15 | 1.10~4.19 | 0.02 | Calf circumference* | 0.923 | 0.840~1.015 | 0.097 |
| Balance(＞25.5cm, ref vs At risk(＜25.4cm) | 2.99 | 0.37~23.99 | 0.3 | Balance* | 0.949 | 0.903~0.998 | 0.040 |
| Flexibility (in the normal, ref vs Below the normal) | 2.04 | 0.74~5.62 | 0.17 | Flexibility* | 0.971 | 0.945~0.997 | 0.032 |
| Muscular endurance (times) | 0.98 | 0.93~1.03 | 0.41 | Muscular endurance (times) | 0.987 | 0.933~1.044 | 0.638 |
| Cardiorespiratory endurance (steps) | 0.97 | 0.96~0.99 | 0.001 | Cardiorespiratory endurance (steps) | 0.976 | 0.960~0.992 | 0.004 |

*MNA, Calf circumference, Balance, Flexibility are used the continuous scale
